# Role of RNA polymerase III transcription and regulation in ischaemic stroke

**DOI:** 10.1080/15476286.2024.2409554

**Published:** 2024-10-03

**Authors:** Chi Kwan Tsang, X.F. Steven Zheng

**Affiliations:** a Clinical Neuroscience Institute, The First Affiliated Hospital of Jinan University, Guangzhou, China; b Rutgers Cancer Institute, The State University of New Jersey, New Brunswick, NJ, USA; c Department of Pharmacology, Robert Wood Johnson Medical School, Rutgers, The State University of New Jersey, Piscataway, NJ, USA

**Keywords:** mTOR, MAF1, RNA polymerase III, ischaemic stroke, acute phase neuroprotection, recovery phase neural repair

## Abstract

Ischaemic stroke is a leading cause of death and life-long disability due to neuronal cell death resulting from interruption of glucose and oxygen supplies. RNA polymerase III (Pol III)-dependent transcription plays a central role in protein synthesis that is necessary for normal cerebral neuronal functions, and the survival and recovery under pathological conditions. Notably, Pol III transcription is highly sensitive to ischaemic stress that is known to rapidly shut down Pol III transcriptional activity. However, its precise role in ischaemic stroke, especially during the acute and recovery phases, remains poorly understood. The microenvironment within the ischaemic brain undergoes dynamic changes in different phases after stroke. Emerging evidence highlights the distinct roles of Pol III transcription in neuroprotection during the acute phase and repair during the recovery phase of stroke. Additionally, investigations into the mTOR-MAF1 signalling pathway, a conserved regulator of Pol-III transcription, reveal its therapeutic potential in enhancing acute phase neuroprotection and recovery phase repair.

## Introduction

Stroke, defined as prolonged acute neurological dysfunction resulting from ischaemia or haemorrhage lasting over 24 h, is a global leading cause of death and disability [1]. Ischaemic stroke, the most prevalent type, arises from reduced blood flow due to thrombosis, embolism, or blood vessel narrowing. Thrombosis involves clot formation within the atherosclerotic arteries, while embolism stems from clots formed elsewhere in the body that travel to the brain, causing vessel blockage [1,2]. Neuronal cells are particularly susceptible to ischaemia. Ischaemia causes oxygen and nutrient deprivation, triggering an ischaemic cascade leading to cell death if blood flow is not promptly restored through reperfusion therapy [3]. Intravenous thrombolysis and endovascular thrombectomy have significantly improved outcomes, but effective neuroprotection methods are still limited [4]. Immediate after an ischaemic stroke, the brain tissue in the infarct core experiences cerebral blood flow arrest, causing rapid cell death. However, the surrounding region of the infarct core, called ‘penumbra’, is salvageable. It has become a focus of neuroprotective therapy after novel biomarkers for penumbra have been identified recently by our research group and others [5–9].

**Figure 1.**
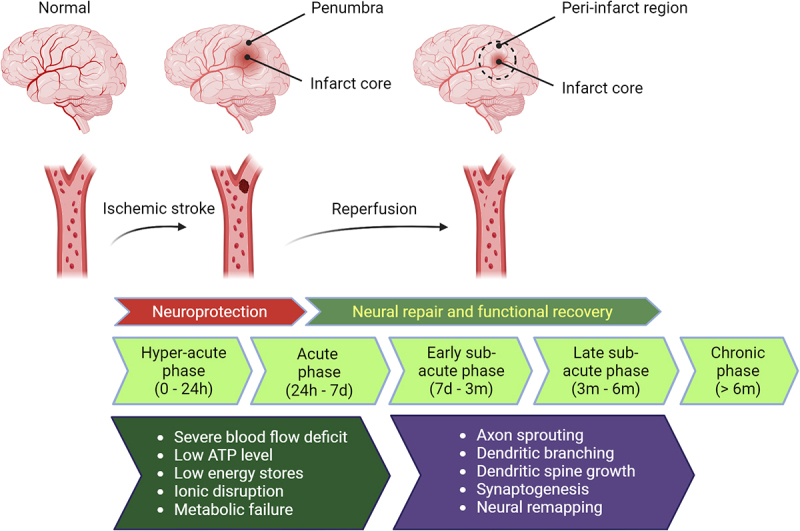
The events and phases after cerebral ischaemic stroke. Ischaemic core consists of necrotic and apoptotic cells in hyper-acute phase. Surrounding region of infarct core is penumbra where cells are still rescuable if reperfusion is possible in a timely fashion. After acute phase, ischaemic core size is stabilized and penumbra will disappear while the peri-infarct region (denoted by the dotted circle) around the infarct core is the major region for spontaneous neural repair and functional recovery which could be further enhanced by medical interventions. Figure created with BioRender.com.

As shown in Figure 1, stroke phases include hyperacute, acute, early sub-acute, late sub-acute, and chronic phases. The neuroprotective therapeutic window primarily lies in the hyper-acute and acute phases to rescue the penumbra tissue [10], in which cells activate adaptive stress responses. Ischaemic cells in the penumbra often exhibit excessive ROS production in mitochondria, leading to oxidative stress during the acute phase [3]. Beyond a critical point, irreversible damage occurs, resulting in a stabilized size of the infarct core after the acute phase. After this neuroprotective window, stroke treatment strategies are shifted to promoting structural changes for neural circuit reconnection and remapping, focusing on neurological recovery, particularly in the peri-infarct region, for restoration of the lost neurological functions (Figure 1). During this neural repair phase, improvement could occur by spontaneous recovery, which could be further boosted by therapeutic interventions mainly in the peri-infarct region. Usually, the recovery rate plateaus in the sub-acute phases, and no more significant functional recovery could be observed in the chronic phase of stroke [4,11,12]. Therefore, it is crucial to identify the effective drug therapy for boosting stroke recovery during the critical period. Figure 1 summarizes ischaemic stroke phases and dynamic molecular and cellular changes in the brain. Recent studies have shown that RNA polymerase III (Pol III) transcriptional activity is highly sensitive to stroke and modulating Pol III affects the outcomes of acute and chronic phases of stroke, suggesting that Pol III is a potentially useful therapeutic target. The goal of this review is to introduce new approaches for stroke recovery.

## Pol III’s transcriptional and cellular functions

**Figure 2.**
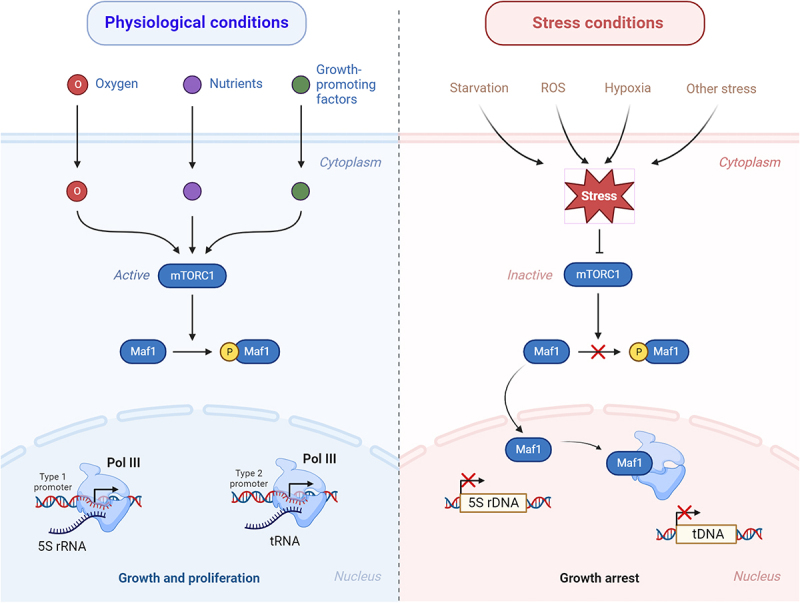
A simplified diagram showing the regulation of Pol III by mTORC1-MAF1 pathway. Mammalian/mechanistic target of rapamycin complex 1, mTORC1; RNA polymerase III, Pol III; transfer RNA, tRNA; ribosomal RNA, rRNA; ROS, reactive oxygen species. Arrow and T-shaped line represent activation and inhibition, respectively. Phosphorylation is denoted by the yellow circle with a ‘P’. See text for detailed description. Figure created with BioRender.com.

Pol III is one of the three primary RNA polymerases in eukaryotes, featuring a conserved core complex and eight constitutive regulatory subunits [13]. This enzyme plays a crucial role in transcribing genes that encode short, non-translating RNAs, including transfer RNAs (tRNA), 5S ribosomal RNA (rRNA), spliceosomal U6 small nuclear RNA (snRNA), and signal recognition particle 7SL RNA [14]. There are three main types of Pol III promoter structures: Type 1 and 2 promoters are located within coding genes, whereas type 3 promoters are located in the 5′ flanking region. The recruitment of Pol III to its target genes is facilitated by specific transcription factors. In Type 1 promoters, Pol III is recruited to TF-IIIA, TF-IIIC, and BRF1/TF-IIIB, consisting of BDP1, BRF1, and TBP, whereas TF-IIIA is absent in Type 2 promoters. In Type 3 promoters, Pol III recruitment is orchestrated by the snRNA-activating protein complex and BRF2–TF-IIIB [15]. The predominant Pol III transcription products, 5S rRNA and tRNAs, are transcribed by Type I and Type II promoters, respectively (Figure 2). We will here focus on how two major Pol III-target genes, 5S rRNA and tRNA, respond to stress conditions.

5S rRNA plays a pivotal role in the functional assembly of the cytoplasmic ribosomes [16]. Its significance lies in guiding the formation of the functional peptidyl transferase centre, a critical aspect of assembling the large ribosomal subunit [16]. Eukaryotic ribosomes consist of a 40S and a 60S subunit. The maturation process of ribosomes entails the coordinated expression of four distinct rRNAs and approximately 80 unique ribosomal proteins (RPs). The 40S subunit is constructed from 30 distinct RPs and an 18S rRNA, while the 60S subunit comprises of 49 unique RPs and single copies of 28S, 5.8S, and 5S rRNA in mammalian cells. Notably, all rRNAs are transcribed by Pol I, with the exception of 5S rRNA that is transcribed by Pol III. Ribosomes are abundantly present in neurons, as evidenced by Nissl staining in brain tissue sections [17]. Ribosomes are responsible for translating mRNAs, determining the protein synthetic capacity of a cell [18,19]. Thus, the regulation of ribosome biogenesis in a cell is crucial for optimizing energy consumption efficiency and adaptation to changing environmental conditions [20]. Furthermore, it has been demonstrated that in the penumbra, ischaemic neurons are salvageable potentially due to the disintegration of endoplasmic ribosomes and Nissl bodies for the rapid suppression of protein synthesis, leading to the reduction of unnecessary energy consumption during ischaemic stress in penumbra [21].

The abundance of ribosomes does not always directly correlate with the rate of protein synthesis. Studies have revealed that mature ribosomes can undergo degradation through autophagy in response to nutrient starvation in *Saccharomyces cerevisiae*, a process facilitated by the Ubp3/Bre5p ubiquitin protease [22]. This selective form of autophagy is termed ‘ribophagy’. Subsequent investigations in mammalian systems have demonstrated the conservation of ribophagy in human embryonic kidney cells, where the nuclear fragile X mental retardation-interacting protein 1 (NUFIP1) acts as a ribosome receptor, facilitating the transfer of ribosomes to autophagosomes. Notably, starvation-induced ribophagy has been shown to promote cell survival during nutrient-deprived conditions [23]. Our recent studies also revealed a key role of autophagy in ischaemic and stressed brain [8,24,25]. These findings imply that the excessive production of ribosomes during normal growth conditions would play a positive role in supporting cell survival during prolonged periods of nutrient stress. This support is achieved by ribophagy which degrades the excessive ribosomes for providing a nutrient source, particularly nucleosides, for energy metabolism essential for maintaining viability and cellular homeostasis [23]. However, whether ribophagy plays a significant role in neurons under ischaemic stroke conditions remains an area for further investigation.

In addition to 5S rRNA and ribosome production, stress can influence cellular tRNA levels through Pol III-mediated transcriptional regulation. The turnover of tRNAs allows cells to adjust the tRNA pool, influencing selective mRNA translation efficiency in response to stress [26]. Due to their intricate structure and chemical modifications, quantifying these tRNA molecules is more challenging compared to other RNAs. Recent advancements in detecting cellular tRNA populations have unveiled that Pol III-dependent changes in tRNA pools can reprogram gene expression patterns based on codon usage in response to environmental conditions. In *S. cerevisiae*, studies have demonstrated dynamic changes in the tRNA pool under diverse stress conditions, facilitating the selective translation of stress-related mRNAs and potentially increasing the production of necessary proteins during stress [27]. Further exploration of ischaemic stroke-induced alterations in Pol III-mediated transcription and tRNA degradation for determining the tRNA pool could offer novel insights into the role of tRNA in mediating global and specific protein synthesis. This suggests the possibility that after an ischaemic stroke, Pol III may adjust the relative abundance of tRNAs based on codon usage, promoting the translation of specific stroke-induced proteins as an adaptive response.

Apart from altering the tRNA pool, stress can induce the formation of tRNA fragments known as tRNA halves or tiRNAs. It has been reported that oxidative stress induces the activation of a specific RNA nuclease called angiogenin, which in turn cleaves the anticodon loop of selective tRNAs to form tRNA fragments, resulting in suppression of global protein synthesis [28]. Recent discovery further revealed that these tRNA fragments possess previously unknown functions, including the regulation of mRNA translation, stress granule formation, epigenetic modifications, cell viability, gene silencing, transposable element (TE) and inflammation [29]. For example, a recent study analysed the whole blood samples collected from patients at 2 days after ischaemic stroke onset, and found that the blood levels of tRNA fragments were increased. Among them, six tRNA fragments were demonstrated to be involved in rebalancing of acetylcholine signalling in CD14^+^ monocytes and modulating post-stroke immunosuppression [30]. In a focal transient cerebral ischaemia-reperfusion model in rats, ischaemic stress induces expression of tRNA modifying enzymes in the brain tissue, and their expressions were correlated with the formation of tRNA fragments. These results suggest that tRNA fragments could be used as biomarkers for stroke [31].

In a rat brain ischaemic model, tRNA^Val^ and tRNA^Gly^-derived fragments were found to be increased in ischaemic brain tissue. In particular, the expression of these tRNA fragments in endothelial cells play a role in inhibition of angiogenesis [32]. A subsequent clinical study analysing the plasma tRNA fragment levels in patients with ischaemic and haemorrhagic stroke revealed that the plasma level of tRNA fragments is correlated with their infarct size and functional outcome, suggesting that its potential to be the biomarker for stroke-induced brain damage and prognosis of stroke patients [33]. Moreover, it has been reported that oxidative stress induces the formation of tRNA^Tyr^ fragment, which is not generated by angiogenin, but derived from pre-tRNA^Tyr^ actively transcribed by Pol III, and these tRNA fragments sensitize motor neuron to oxidative stress-induced cell death [34]. Subsequent study further verified that certain Pol III-derived tRNA fragments are neurotoxic and can cause neuronal necrosis [35]. Therefore, the roles of tRNA fragments seem to be context-dependent and more research works are need to clarify their pathophysiological functions in ischaemic stroke. In this review, we will focus on the role of Pol III transcription and its regulation in different phases after ischaemic stroke. We will also discuss the therapeutic potential of targeting Pol III transcription for the treatment of ischaemic stroke.

## mTOR-MAF1 signalling in the regulation of Pol III transcription

As mentioned before, genes transcribed by Pol III play a pivotal role in cellular growth, encompassing crucial processes such as ribosome biogenesis, pre-mRNA splicing, and protein synthesis and transport. These processes are highly energy-consuming and contribute to normal physiological functions under favourable conditions. It is estimated that Pol III transcription, coupled with its coregulated Pol I activity, is responsible for synthesizing up to 85% of total cellular RNA in actively growing cells [36,37]. To adapt to changing environmental conditions, particularly nutrient availability, cells must dynamically regulate Pol III transcription. The mechanistic target of rapamycin (mTOR) complex 1 (mTORC1) serves as a central component in nutrient signalling [19,38–40]. It is a serine – threonine kinase and the molecular target of the immunosuppressive and anticancer drug rapamycin [41]. mTORC1 is a major regulator of Pol III transcription. In various cell types and organisms, ranging from yeast to humans, the inhibition of mTORC1 by rapamycin treatment or nutrient starvation, results in a swift down-regulation of Pol III transcription [18,19,38,42–46] (Figure 2).

MAF1 is a major downstream effector in mTORC1 regulation of Pol III transcription [47]. MAF1 was originally identified in *S. cerevisiae* that later shown to be an evolutionarily conserved transcriptional repressor of Pol III [48,49]. When activated by nutrients, mTORC1 directly phosphorylates and inhibits MAF1, stimulating Pol III transcription and cellular growth [48,49]. Under stress conditions such as starvation, MAF1 becomes dephosphorylated, entering into the nucleus and repressing Pol III transcription [46,50–52] (Figure 2). Yeast Maf1 binds to the RPC34 subunit of Pol III complex, the active site required for binding with the transcription initiation factor Brf1 (TF-IIIB), preventing formation of the pre-initiation complex at Pol III promoters [53,54]. MAF1 also prevents Pol III transcription re-initiation [53]. Interestingly, MAF1 displays selectivity towards distinct Pol III-transcribed genes. A subset of tRNA genes exhibits low responsiveness to MAF1 repression upon stressed conditions, which consist of at least one tRNA isoacceptor for each of the 20 amino acids [55,56]. Notably, emerging evidence has shown that mTOR is not only found in the cytoplasm but also in the nucleus across a broad spectrum of cell types. Intriguingly, mTOR has been found to be physically associated with a diverse array of Pol I- and Pol III-transcribed genes, and mTOR occupancy on the promoter of Pol III-transcribed genes is highly sensitive to rapamycin treatment [57]. Whether and how mTOR and MAF1 interacts as a complex at chromatin during transcriptional modulation in neurons and other cell types remains to be elucidated.

More recently, accumulating evidence has shown that MAF1 also regulates certain Pol II transcribed genes that encode for proteins [58]. Johnson and colleagues first discovered that MAF1 binds to the promoter of TATA-binding protein (TBP) gene at the Elk-1 binding motif in glioblastoma for inhibiting Pol II-mediated TBP transcription [59]. Subsequent studies showed that overexpression of MAF1 not only inhibits Pol-III transcription but also represses genes involved in lipid metabolism such as *FASN* and *ACC1* which are transcribed by Pol II [60]. In *Maf1* knockout mice, expression of nicotinamide N-methyltransferase in liver and muscle cells is decreased compared with wild-type control [61]. Deletion of *Maf1* in yeast results in the repression of *FBP1* and *PCK1*, the two major genes controlling gluconeogenesis [62]. Our recent studies in liver cancer models in vitro and in vivo further revealed that overexpression of MAF1 inhibits tumour cell growth through direct binding to the promoter of PTEN. Furthermore, the binding of MAF1 leads to enhanced acetylation level at the PTEN promoter, and upregulation of PTEN expression. Our results indicate that MAF1 can serve as an activator of Pol II-transcribed PTEN gene for inhibition of AKT-mTOR signalling activity through a feed-forward mechanism [43]. Recent findings by us and Xiang’s group using the Cleavage under targets and tagmentation (CUT&Tag) approach for mapping the whole-genome MAF1 binding sites in cerebral cortical neurons and retinal cells further unveiled that MAF1 regulates expression of a large set of Pol II-transcribed genes involved in survival, differentiation and neural repair, in addition to its well-recognized Pol III transcription [45,63].

## Role of MAF1 in stress resistance and survival under acute phase of ischaemic stroke

MAF1 has emerged as a conserved regulator of stress responses for restricting the high-energy consuming Pol III transcription and protein synthesis, which is essential for conserving cellular resources to support survival. Loss of *MAF1* function results in decreased ability to confer stress resistance in various organisms [47]. In budding yeast, Maf1 is required for repressing Pol III and maintaining fitness under a wide variety of stress conditions [64,65]. In *Arabidopsis*, Maf1-mediated repression of Pol III transcription is critical for cell survival during environmental stress through regulation by the TOR pathway. *maf1* deleted *Arabidopsis* exhibits elevated Pol III transcription of 5S rRNA and pre-tRNAs in leaf cells, reducing cell survival under diverse environmental stresses [66].

MAF1’s role in repressing energy-consuming Pol III-dependent transcription suggests that it is pro-survival during stress conditions, such as the acute phase of stroke. However, paradoxically, some reports indicate an opposite role of MAF1 in stress responses. MAF1 has been shown to bind to the PTEN promoter and activate PTEN transcription, suppressing mTORC1 signalling activity as a feed-forward mechanism in liver cancer cells [43]. Similar mechanisms have been identified in neurons [67,68]. Notably, mTOR signalling plays a crucial role in the survival of retinal ganglia cells (RGC), which is counteracted by PTEN, a negative regulator of mTORC1 [69–73]. Knockdown of *Maf1* in RGC enhances cell survival after optic nerve crush (ONC), evidenced by a thicker retinal ganglion cell complex (GCC) in *Maf1*-knockdown mice [67]. This *in vivo* evidence supports the protective role of inhibiting MAF1 against retinal ganglion cell injury. The plausible explanation for this observation is that MAF1 may mediate cell viability in a Pol III-independent manner. Indeed, emerging evidence has shown that MAF1 not only regulates Pol III transcription but also many Pol II-transcribed gene, in particular PTEN. Activated MAF1 may promote PTEN expression in RGC after injury, leading to suppression of mTOR signalling pathway and/or other survival signalling such as AKT which is downstream of PTEN [74]. PTEN is also known to play a negative role in RGC viability after optic nerve injury [75]. Therefore, it is possible that knockdown of *Maf1* mitigates the PTEN-mediated inhibition of survival signalling activity after injury, leading to the observed enhanced viability. Alternatively, knockdown of *Maf1* may abolish other Pol II-transcribed genes that play a regulatory role in apoptosis and thus loss of *Maf1* confers elevated viability in these cells. Furthermore, the survival effect of MAF1-modulated Pol II and Pol III transcription is likely to be cell type- and context-dependent, as well as differs under various pathophysiological conditions.

In line with this observation, a recent study also demonstrated a negative role of MAF1 in a mouse ischaemic stroke model, in which downregulation of MAF1 by miRNA-122 protects neurons against ischaemic damage [76]. However, the authors did not investigate the change of Pol III transcription under this condition and the underlying molecular explanation was not addressed in this study. Although compelling evidence has indicated that MAF1 plays a positive role in conserving the intracellular energy resource through repression of Pol III transcription, ischaemic stroke-induced brain damage could be complicated and energy metabolic reservation is only one of the factors contributing to cell viability. In addition, the complex dynamic nature of post-stroke immune responses could influence the outcome resulted from *MAF1* knockdown, and therefore energy conservation may not be the only key factor for the determination of cell survival. It is plausible that one of the consequences of MAF1 downregulation may include inactivation of mTORC1 signalling through the feedback mechanism and thus compromising the cell viability. Furthermore, the off-target effect by miRNA-122 may promote survival under the tested condition. Downregulation of MAF1 may also alleviate the survival inhibitory activity of PTEN pathway or via other yet-to-be identified MAF1 target genes involved in survival regulation. More work is needed to clarify the role of MAF1 and the underlying mechanisms in ischaemic stroke conditions.

Indeed, in certain stress conditions, MAF1 appears to have a dual role in cell viability. For instance, in *C. elegans*, MAF1-dependent intracellular lipid accumulation correlates positively with UV-induced DNA damage. Genetic ablation of MAF1 activates DNA damage response, enhancing survival [77]. Given that ischaemic stroke could induce DNA damage through oxidative stress, *MAF1* knockout/knockdown might exacerbate oxidative damage in neurons by enhancing lipid peroxidation, necessitating investigation into potential alterations in Pol III transcription in this context. Another *C. elegans* study reveals that *maf1* deletion enhances stress tolerance by boosting oxidative stress response, mitochondrial unfolded protein response, and autophagy, thereby improving survival and extending lifespan through reduced tRNA synthesis due to decreased mTOR activity [78]. Intriguingly, *maf1* deletion alleviates paralysis in a *C. elegans* model of Alzheimer’s disease [78]. Furthermore, whole-body knockout of *Maf1* in female mice results in improved survival and extended lifespan, associated with increased autophagy [61]. These studies collectively highlight the positive and negative roles of MAF1 in cellular survival across diverse organisms and tissues. To date, robust data are still lacking on the role of MAF1 in ischaemic stroke. Further studies are warranted to delineate a detailed MAF1-mediated mechanism in the acute phase of ischaemic stroke in appropriate animal models.

## Role of MAF1 in neural repair and functional recovery during the recovery phase of ischaemic stroke

The protein synthetic capacity of neurons is pivotal for neurite development and regeneration [60,79]. In developing or repairing neurons, ribosome and tRNA synthesis support the increasing demand for protein synthesis, crucial for axonal extension, dendritic branching, and neuronal reconnection [80]. Nissl bodies undergo fragmentation after axonal injury or neuronal death, underscoring the close involvement of ribosomes in axonal repair [81]. Notably, MAF1 is highly expressed in the cerebral cortex, hippocampus, and retina neurons [67,68], suggesting that MAF1-regulated Pol III transcription is important in these brain domains. *Maf1* silencing promotes dendritic spine growth and branching in cultured hippocampal neurons *in vitro*, while *Maf1* overexpression has the opposite effect [68]. *In vivo*, MAF1 negatively regulates dendritic spine growth and synaptic density in mouse hippocampal tissues, impacting learning and memory in young adult mice (6 weeks) [68]. In an optic nerve injury model, *Maf1* knockdown in retinal ganglion cells promotes axon regeneration after optic nerve crush [67]. In cultured mouse primary cortical neurons, MAF1 mediates mTOR regulation of Pol III-dependent transcription of rRNA and tRNA genes [45]. *Maf1* knockdown enhances neurite outgrowth, while *Maf1* overexpression inhibits neurite outgrowth, establishing MAF1 as an intrinsic negative regulator of Pol III transcription and neurite outgrowth in cortical neurons.

In a mouse *in vivo* stroke model, MAF1 was investigated in peri-infarct neurons during spontaneous recovery after ischaemic stroke. MAF1 protein expression was notably increased, primarily in the dephosphorylated/active form during the first two weeks after stroke in the peri-infarct cortex compared to the sham control [45]. Because this is a period crucial for spontaneous neural repair, the elevated MAF1 expression suggests that it has a repressive role in Pol III transcription in the peri-infarct tissue. Under ischaemic conditions in the peri-infarct microenvironment, MAF1 activation results in a reduction in the energy-consuming processes like Pol III-dependent transcription, ribosome biogenesis, and protein synthesis. In the meanwhile, this response also inhibits neurite growth and axon regeneration processes. Consistently, *Maf1* knockdown in the peri-infarct cortex led to increased Pol III transcription, ribosomal biogenesis, global protein synthesis, axon regeneration, dendritic arborization, and significantly enhanced recovery of neurological functions compared to control mice [45]. Interestingly, MAF1 not only represses the transcription of Pol III-transcribed genes but also CREB-dependent genes crucial for memory, learning, neurodevelopment, and neuroplasticity [45]. Collectively, these results demonstrate that MAF1 acts as an intrinsic suppressor against spontaneous neural repair and functional recovery after ischaemic stroke. They further suggest that MAF1 could be a potential therapeutic target for enhancing functional recovery after ischaemic stroke and other CNS injuries.

**Figure 3.**
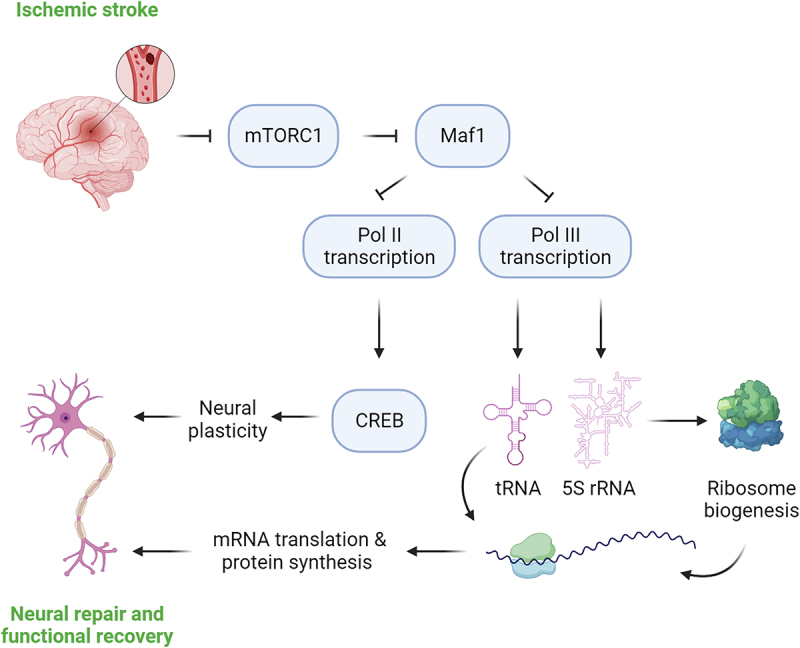
Role of MAF1 in neural repair and restoration of neurological functions in recovery phase. Mammalian/mechanistic target of rapamycin complex 1, mTORC1; RNA polymerase I, Pol I; transfer RNA, tRNA; ribosomal RNA, rRNA; cyclic AMP response element binding protein, CREB. Figure created with BioRender.com.

In an *in vivo* permanent stroke model, MAF1 is notably upregulated in the peri-infarct tissue of the cerebral cortex during the critical repair period of 7 to 14 days after stroke onset, gradually returning to background levels at 2 months. This suggests that the microenvironment around the peri-infarct tissue, with compromised blood supply and a negative growth inhibitory environment in the infarct core region, may sustain mTOR suppression and activate MAF1. In the acute phase of stroke, this appears to be an adaptive mechanism for brain neurons to resist ischaemic stress. However, during the recovery phase, especially when nutrients and oxygen remain undersupplied in the peri-infarct cortical tissue, active MAF1 repression of Pol III transcription limits axon regeneration, dendritic branching, and functional recovery. Targeting MAF1 through small molecule inhibitors, AAV-mediated knockdown, or RNA-based nanoparticle delivery could be a useful approach for stroke management during the recovery phase (Figure 3).

## Future perspectives

This review focuses on the mechanism of mTORC-MAF1 signalling in regulating Pol III-dependent transcription, as well as its physiological and pathological roles under ischaemic conditions. While some progress has been made in understanding Pol III transcription in CNS neurons, little is known about its roles in other brain cells such as astroglia, microglia, oligodendrocytes, cerebral vascular cells, and infiltrated immune cells, especially during the acute and chronic phases of stroke. These non-neuronal cells have also been implicated in the pathogenesis of ischaemic stroke. Considering the impact of Pol III transcription on cell proliferation and growth, exploring its role in proliferating glial and vascular cells could lead to new insights into the cellular and molecular mechanisms of ischaemic stroke-induced injury and spontaneous recovery, which may present new therapeutic opportunities targeting the acute and/or chronic recovery phases.

It’s noteworthy that unconditional *Maf1* knockout in mice doesn’t affect normal development or promote tumorigenesis [61]. Instead, it provides health benefits such as resisting high-fat diet-induced liver steatosis and obesity [61], suggesting MAF1 as an excellent therapeutic target for preventing and treating cerebral ischaemic stroke. Further studies are needed to test the efficacy and safety of targeting MAF1 using methods such as AAV- or nanoparticle-mediated delivery of interfering RNAs [18]. Another area of interest is to identify the small molecules that could manipulate Pol III-dependent transcription or MAF1 activity by specific activators and inhibitors which could be used as therapeutic drugs in acute and chronic phases of ischaemic stroke. Notably, a cell-permeable indazolo-sulphonamide compound ML-60218 (CAS 577784-91-9) has been developed as a selective inhibitor against Pol III-transcription [82]. ML-60218 has been shown to be able to suppress yeast cell growth by repressing Pol III-mediated tRNA transcription [82]. It would be interesting to test whether it possesses therapeutic effect on acute phase of ischaemic stroke in future studies. More pre-clinical studies in different stroke models are warranted to explore its therapeutic potential in the acute phase of stroke.

For chronic phase of stroke, the use of Pol III activator would likely offer beneficial effect for enhancing the neural repair in post-stroke recovery phase. However, neither specific small molecules of Pol III activator nor MAF1 inhibitor is currently available. Thus, the identification of small molecules selectively inhibiting MAF1 could be an attractive approach. To achieve this goal, the state-of-the-art approach such as AI-assisted protein folding analysis such as AlphaFold, RoseTTA and molecular docking approach is encouraged in future studies.

In addition to Pol III, ischaemic stress affects Pol I and Pol II activities. It has been shown that a wide variety of cellular stresses attenuates Pol I-dependent rRNA transcription [83]. Therefore, it is anticipated that in response to nutrient starvation and hypoxic stress resulting from an ischaemic stroke, the Pol I-transcribed genes 5.8S, 18S and 28S ribosomal RNAs and Pol II-transcribed ribosomal protein genes are likely to be downregulated. It has been demonstrated that the intracellular energy imbalance resulting from glucose deprivation leads to inhibition of Pol I-dependent rRNA transcription through the establishment of silent chromatin in the rDNA loci [84]. Stress-induced inactivation of UBF and TIF-IA, two key basic transcriptional factors of Pol I has also been reported [83].

Pol II-dependent transcriptional repression of ribosomal protein genes is another prominent cellular response to various stress conditions [85]. In *S. cerevisiae*, ribosomal protein genes are regulated mainly at the transcriptional level by Pol II with many transcription factors. In higher eukaryotes, how the coordinated regulation of ribosomal protein genes remains incompletely understood. The highly conserved nutrient sensor mTOR pathway is the most well-studied signalling pathway thought to be responsible for the coordinated transcriptional regulation of ribosomal protein genes as well as the rRNA components for governing the homeostatic ribosome production in response to stress conditions [19]. Currently, how ischaemic stress affects Pol I-transcribed rRNAs and Pol II-transcribed ribosomal protein genes in brain cells remain unknown. Future studies are necessary to clarify their precise roles and evaluate their therapeutic potential for stroke, especially during the chronic phase of ischaemic stroke for neural repair and functional recovery. Because MAF1 plays a key role in the regulation of ribosomal genes as well as certain stress-responsive Pol II-transcribed genes, it will be important to have an in-depth understanding of mTOR-MAF1 signalling axis in control of ischaemic stress response, which could lead to novel treatment strategies for stroke.

## Data sharing plan

Data sharing is not applicable to this article as no new data were created or analysed in this study.
